# In-Depth Transcriptome Analysis of the Red Swamp Crayfish *Procambarus clarkii*

**DOI:** 10.1371/journal.pone.0110548

**Published:** 2014-10-22

**Authors:** Huaishun Shen, Yacheng Hu, Yuanchao Ma, Xin Zhou, Zenghong Xu, Yan Shui, Chunyan Li, Peng Xu, Xiaowen Sun

**Affiliations:** 1 Key Laboratory of Freshwater Fisheries and Germplasm Resources Utilization, Ministry of Agriculture, Freshwater Fisheries Research Center, Chinese Academy of Fishery Sciences, Wuxi, China; 2 Wuxi Fisheries College, Nanjing Agricultural University, Nanjing, China; 3 The Center for Applied Aquatic Genomics, Chinese Academy of Fishery Sciences, Beijing, China; Fish Vet Group, Thailand

## Abstract

The red swamp crayfish *Procambarus clarkii* is a highly adaptable, tolerant, and fecund freshwater crayfish that inhabits a wide range of aquatic environments. It is an important crustacean model organism that is used in many research fields, including animal behavior, environmental stress and toxicity, and studies of viral infection. Despite its widespread use, knowledge of the crayfish genome is very limited and insufficient for meaningful research. This is the use of next-generation sequencing techniques to analyze the crayfish transcriptome. A total of 324.97 million raw reads of 100 base pairs were generated, and a total of 88,463 transcripts were assembled *de novo* using Trinity software, producing 55,278 non-redundant transcripts. Comparison of digital gene expression between four different tissues revealed differentially expressed genes, in which more overexpressed genes were found in the hepatopancreas than in other tissues, and more underexpressed genes were found in the testis and the ovary than in other tissues. Gene ontology (GO) and KEGG enrichment analysis of differentially expressed genes revealed that metabolite- and immune-related pathway genes were enriched in the hepatopancreas, and DNA replication-related pathway genes were enriched in the ovary and the testis, which is consistent with the important role of the hepatopancreas in metabolism, immunity, and the stress response, and with that of the ovary and the testis in reproduction. It was also found that 14 vitellogenin transcripts were highly expressed specifically in the hepatopancreas, and 6 transcripts were highly expressed specifically in the ovary, but no vitellogenin transcripts were highly expressed in both the hepatopancreas and the ovary. These results provide new insight into the role of vitellogenin in crustaceans. In addition, 243,764 SNP sites and 43,205 microsatellite sequences were identified in the sequencing data. We believe that our results provide an important genome resource for the crayfish.

## Introduction

The red swamp crayfish *Procambarus clarkii* is a freshwater crayfish species that is native to parts of Mexico and the United States [1], but is also commonly found outside its natural range in Asia, Africa, Europe, and elsewhere in the Americas, where it is often considered to be an invasive pest [2]. *P. clarkii* was introduced to China from Japan in the 1930s [3]. Crayfish farming began in in the 18th century in Louisiana in the USA, where the species was cultivated in rice fields. Crayfish have been farmed extensively in China since the 1990s, and China is now the world's leading crayfish producer [4].

*P. clarkii* is a highly adaptable, tolerant, and fecund freshwater crayfish that can inhabit a wide range of aquatic environments, including those with moderate salinity, low oxygen levels, extreme temperatures, and pollution [2], [5]. Because of these characteristics, in addition to its economic role, the crayfish has become an important crustacean model organism in research on viral infection [6]–[10], animal behavior [11]–[17], and environmental stress and toxicity [18]–[23].

Despite great interest in this organism, knowledge of the crayfish genome is very limited, and gene discovery has been performed on a relatively small scale. Only 330 expressed sequences (EST) and 547 nucleotide sequences have been deposited in GenBank (accessed on Jul 29, 2014) for the crayfish, which is fewer than the close relative *Pacifastacus leniusculus* (1063 EST and 1100 nucleotide sequences) and far less fewer than other economically important crustaceans, such as the freshwater prawn *Macrobrachium nipponense*, the giant freshwater prawn *Macrobrachium rosenbergii*, the pacific white shrimp *Litopenaeus vannamei*, and others. Furthermore, only a very few genetic markers have been discovered for *P. clarkii* [3], [24]–[26].

The traditional methodology to explore expressed sequence tags (ESTs) involves construction of a cDNA library followed by Sanger sequencing, which is time-consuming and inefficient. Normally, the numbers of ESTs generated using this method is no more than ten thousand [27]. In recent years, next-generation sequencing technologies from companies such as 454 Life Sciences, Illumina, and Applied Biosystems (SOLiD sequencing) have been widely used to explore genomic information in model and non-model organisms. In comparison to traditional Sanger sequencing technology, next-generation sequencing technologies are superior in many aspects, and in general they are able to provide enormous amounts of sequence data with a greater breadth and depth of information, in shorter times and at a significantly lower cost [28]–[30]. The expressed sequences generated using next-generation sequencing technologies are often on the order of thousands or hundreds of thousands of sequences, which are ten-fold or one-hundred-fold greater than the number identified by traditional technologies.

Crustaceans studied using next-generation sequencing technologies include the giant freshwater prawn *Macrobrachium rosenbergii* [31], the orient river prawn *Macrobrachium nipponense* [32], the Chinese mitten crab *Eriocheir sinensis* [33]–[36], the pacific white shrimp *Litopenaeus vannamei* [37]–[40], the Chinese shrimp *Fenneropenaeus chinensis* [41], the pandalid shrimp *Pandalus latirostris* [42], and the crab *Portunus trituberculatus* [43]–[44]. These data have significantly enriched our genetic and genomic knowledge of crustaceans.

In this study, hi-seq sequencing technology was used to sequence the transcriptomes of 4 major organs in the crayfish: hepatopancreas, muscle, ovary, and testis. This data was used to generate expressed sequence data, simple sequence repeat markers, and SNP markers that represent a resource for trait mapping, as well as differential organ gene expression profiles, to better understand the functions of the studied organs in the crayfish. We believe that the data obtained from this study represent an import resource for crayfish research into gene function, molecular events associated with breeding, and other areas.

## Material and Methods

### Ethics statement

This study was approved by the Animal Care and Use Committee of the Center for Applied Aquatic Genomics at Chinese Academy of Fishery Sciences.

### Animal collection

*P. clarkii* weighing approximately 10–20 g were collected from a crayfish farm in Xuyi, Jiangsu Province, China. Collected crayfish were cultured in water tanks with adequate aeration at 20°C and a natural photoperiod, and were fed with a commercial crayfish diet once per day. Four tissue types (hepatopancreas, muscle, ovary, and testis) were collected, and each group of tissues contained samples from approximately ten crayfish. The tissue samples were frozen immediately in liquid nitrogen, and stored at −80°C.

### RNA isolation and Illumina sequencing

Total RNA from various tissues was isolated using the RNeasy Plus Mini Kit (Qiagen, Valencia, CA, USA) according to the manufacturer's protocol, and treated with RNase-free DNase I (Qiagen) to remove genomic DNA. RNA integrity was evaluated by 1.5% agarose gel electrophoresis. RNA concentrations were measured and purity was determined using a NanoDrop ND-1000 spectrophotometer (NanoDrop Technologies, Wilmington, DE, USA).

RNA-seq library preparation and sequencing was carried out by the Genomic Analysis Lab of The Institute of Genetics and Developmental Biology of the Chinese Academy of Sciences (Beijing, China). Approximately 5 µg of DNase-treated total RNA was used to construct a cDNA library following the protocols of the Illumina TruSeq RNA Sample Preparation Kit (Illumina, San Diego, CA 92122, USA). The cDNA libraries were amplified by PCR and contained TruSeq indexes 1–4 within the adaptors. Amplified libraries yielded approximately 500 ng of cDNA with an average length of approximately 270 base pairs (bp). Finally, the libraries were sequenced on an Illumina HiSeq 2000 instrument with 100 bp paired-end (PE) reads.

### *De novo* assembly and transcriptome analysis

Raw reads, which were generated by the Illumina/Solexa sequencer, were first trimmed by removing adapter sequences. Low quality reads (quality scores less than 20) were trimmed and short length reads (<10 bp) were removed [45]–[46]. The resulting high-quality reads were used in subsequent assembly. The Crayfish transcriptome was *de novo* assembled using Trinity software (vision 2013.02.25) with the default parameters [47]. In brief, three steps were performed. First, data was processed by Inchworm, in which the high-quality reads were combined to form longer fragments called contigs. Second, data was processed by Chrysalis, in which sequences were obtained by connecting contigs in such a manner that they could not be extended on either end, which resulted in de Bruijn graphs. Finally, de Bruijn graphs were further treated by Butterfly to obtain transcripts.

### Transcriptome annotation and gene ontology analysis

All transcripts were compared with the NCBI non-redundant (nr) protein database, GO database, COG database, and KEGG database for functional annotation using BLAST software with an e-value cutoff of 1e-5 [48]. Functional annotation was performed with gene ontology (GO) terms (www.geneontology.org) that were analyzed using Blast2go software (http://www.blast2go.com/b2ghome) [49]. The COG and KEGG pathway annotations were performed using Blastall software against the COG and KEGG databases [48].

### Differentially expressed genes

To obtain expression levels for every transcript in different tissues, cleaned reads were first mapped to all transcripts using Bowtie software [50]–[51], then the FPKM (fragments per kilobase of exon per million fragments mapped) value of every transcript was obtained using RSEM (RNASeq by expectation maximization, http://deweylab.biostat.wisc.edu/rsem/) software. Differentially expressed genes were identified using edgeR (empirical analysis of digital gene expression data in R, http://www.bioconductor.org/packages/release/bioc/html/edgeR.html) software [52]–[53]. For this analysis step, the filtering threshold was set as an FDR (false discovery rate) <0.5.

### RT-PCR amplification of transcripts

To validate the assembly of the crayfish transcriptome, 20 selected transcripts were used for expression analysis by RT-PCR. Total RNA was prepared from the four tissues (hepatopancreas, muscle, ovary, and testis) of crayfish using Trizol reagent in accordance with the manufacturer's instructions. Total RNA was treated with RQ1 RNase-free DNase (Promega, Madison, WI, USA) to avoid genomic DNA contamination, and then reverse transcribed using M-MLV reverse transcriptase (Promega, USA) according to the manufacturer's instructions. The synthesized cDNA was used as a template for PCR. The following PCR program was used: denaturation at 94°C for 3 min, 35 amplification cycles of 94°C for 30 s, 58°C for 30 s, and 72°C for 30 s, and a final extension at 72°C for 10 min. The PCR products were determined by 1.5% agarose gel electrophoresis using DNA markers. The expression of the 18S RNA gene of *Procambrus clarkii* (accession number: EU920952.1) was selected as reference gene, using the primer pair *Pc18S*-F (5′-ATCACGTCTCTGACCGCAAG-3′) and *Pc18S*-R (5′-GACACTTGAAAGATGCGGCG-3′).

### SNP and microsatellite sequence identification

The raw reads were exported in FASTQ format to allow them to be imported into software for SNP calling. SAMtools (http://samtools.sourceforge.net/) and VarScan (http://varscan.sourceforge.net/) software were applied to align reads to the reference transcriptome and to detect SNPs [54]–[55]. For this analysis step, the filtering threshold was set as a quality score no less than 20.

Msatcommander software was used to identify microsatellites from assembled contigs, as well as for primer design [56]. The mononucleotide repeats were ignored by modifying the configuration file. The repeat thresholds for di-, tri-, tetra-, penta-, and hexa-nucleotide motifs were set as 8, 5, 5, 5, and 5, respectively. Only microsatellite sequences with flanking sequences longer than 50 bp on both sides were collected for future marker development.

## Results and Discussion

### Illumina sequencing from crayfish tissues

Illumina-based RNA-sequencing (RNA-Seq) was performed with samples of four tissue types from crayfish. A total of 324.97 million paired-end reads were generated with a read length of 100 bp, of which 102.46 million reads were from the hepatopancreas, 83.51 million reads were from muscle, 84.94 million reads were from the ovary, and 36.06 million reads were from the testis. All raw sequence data were deposited in the NCBI Sequence Read Archive (SRA) under accession code SRP044128. After trimming of low quality reads and short reads, a total of 306.73 million high-quality sequences (94.73%) were obtained (Table S1 in File S1), and these sequences were used for further analysis.

### *De novo* assembly of the transcriptome

**Figure 1:**
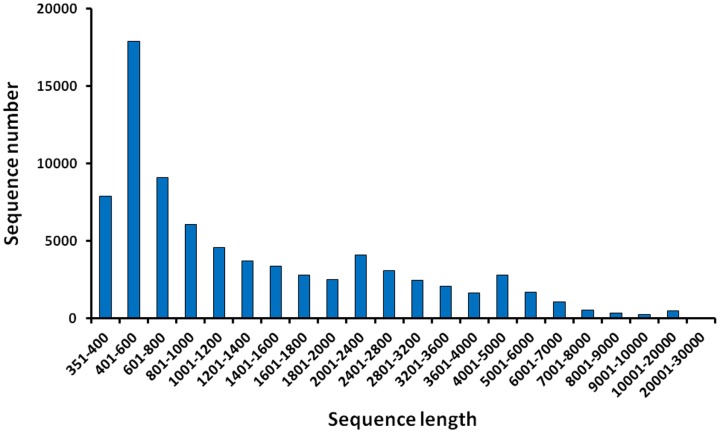
Length distribution of assembled transcripts of *P. clarkii.*

At present, *P. clarkii* has no reference genome sequence, therefore a *de novo* assembly strategy was utilized, in which the crayfish transcriptome was *de novo* assembled by Trinity software (version 2013.02.25) using the default parameters. *De novo* assembly of 306.73 million high-quality sequences generated a total of 88,463 transcripts that ranged from 351 to 34,708 bp in length, with an average length of 1655.49 bp (Table 1). The length distribution of transcripts is shown in Figure 1. Most of the transcripts (23.71%) were 401–600 bp in length, 11.51% ranged from 601–800 bp, and 10.90% ranged from 1–400 bp. These 88,463 transcripts yielded a total of 50,219 non-redundant transcripts because of alternative splicing; therefore, it was possible to match two or more transcripts to one gene. Our sequence data provided a large number of transcripts as compared to publicly available data from the Genbank database, which represent a convenient source of information for future full length cDNA cloning and gene function research in *P. clarkii*. Prior to this study, only 330 EST and 547 nucleotide sequences were listed in the GenBank database. Our study supplied 50,219 non-redundant transcripts, which has significantly enriched our knowledge of the *P. clarkii* genome and will facilitate further study of the functions of *P. clarkii* genes.

**Table 1:** Summary of Illumina Hiseq2000 assembly and analysis of *P. clarkii* transcriptomic sequences.

Type	Number
Total genes	50219
Total transcripts	88463
Total residues	146449732
Average length	1655.49
Largest transcript	34708
Smallest transcript	351

### Functional annotation

Protein coding sequences of transcripts were predicted using a tool supplied by Trinity software (http://trinityrnaseq.sourceforge.net/analysis/extract_proteins_from_trinity_transcripts.html). Of the 88,463 transcripts, 42,905 were found to contain open reading frames (ORFs), with an average protein coding length of 552.78 bp and a mean nucleotide length of 2551.34 bp. These isosequences likely represent genes that play essential roles in *P. clarkii* biological processes.

88,463 transcripts were compared with the NCBI non-redundant (nr) protein database, GO database, COG database, and KEGG database for functional annotation using BlastX with an e-value cutoff of 1e-5 (Table S2 in File S1). A total of 31,763 transcripts (35.91% of all transcripts) had significant hits in at least one of these databases, which corresponded to 11,222 genes (21.63% of all genes). A total of 30,779 transcripts (96.90% of all annotated transcripts) had significant hits in the nr protein database, which corresponded to 10,862 genes (96.79% of all annotated genes). The gene names of top BLAST hits were assigned to each transcript with significant hits, and 3110 transcripts from *P. clarkii* were best matched with genes from *Daphnia pulex*, 2675 transcripts were best matched with genes from *Tribolium castaneum*, and 1837 transcripts were best matched with genes from *Pediculus humanus* (Figure S1). *Daphnia pulex* is a primitive water flea, *Tribolium castaneum* is a type of beetle, and *Pediculus humanus* is a louse species that infests humans. Thus, the genes from *P. clarkii* were most similar to those known from crustaceans and insects, and the distribution of significant BLAST hits over different organisms reflects the phylogenetic relationship between *P. clarkii* and other species.

**Figure 2:**
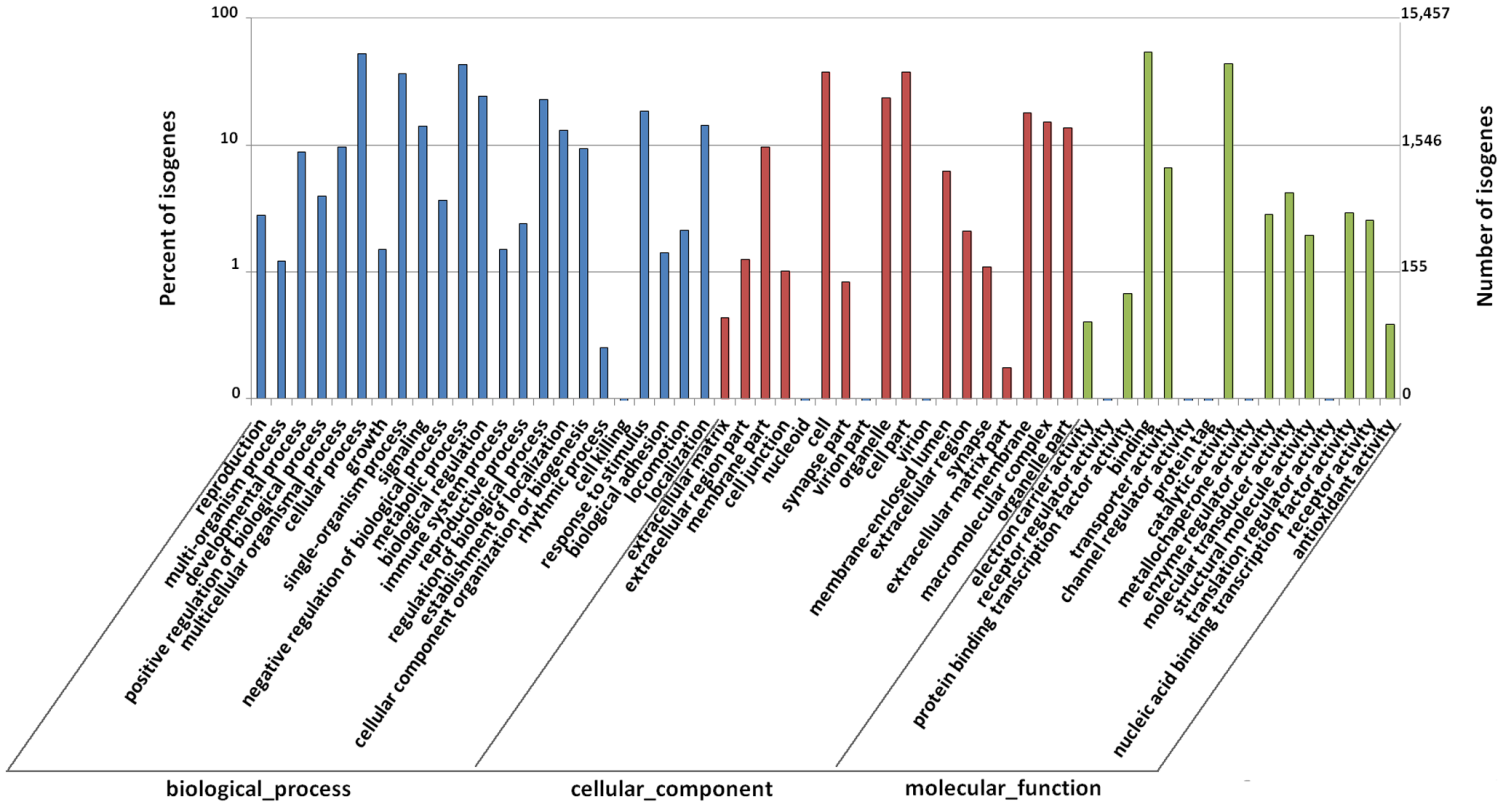
Gene ontology (GO) classification of transcripts of *P. clarkii*. GO terms were processed by Blast2Go and categorized at level 2 under three main categories (biological process, cellular component, and molecular function).

GO analysis was conducted on annotated transcripts using blast2go software. A total of 15,457 transcripts, corresponding to 5890 genes, were assigned at least one GO term for biological processes, molecular functions, and cellular components, and the output of the GO annotations was plotted (Figure 2). Terms from the molecular function term group made up the majority of significant terms (12,842 transcripts, 88.08%), followed by the biological process group (10,241 transcripts, 66.25%) and the cellular component group (7,406 transcripts, 47.91%). For biological processes, genes involved in cellular processes (GO: 0009987, 8,148 transcripts), metabolic processes (GO: 0008152, 6,622 transcripts), and single-organism process (GO: 0044699, 5,623 transcripts) were highly represented. For molecular functions, binding (GO: 0005488, 8,313 transcripts) and catalytic activity (GO: 0003824, 6,781 transcripts) were the most represented GO terms. For cellular components, cells (GO: 0005623, 5,778 transcripts), cell part (GO: 0044464, 5,778 transcripts), and organelles (GO: 0043226, 3,632 transcripts) were the most represented terms. There were 9 identified terms that contained fewer than 10 transcripts.

**Figure 3:**
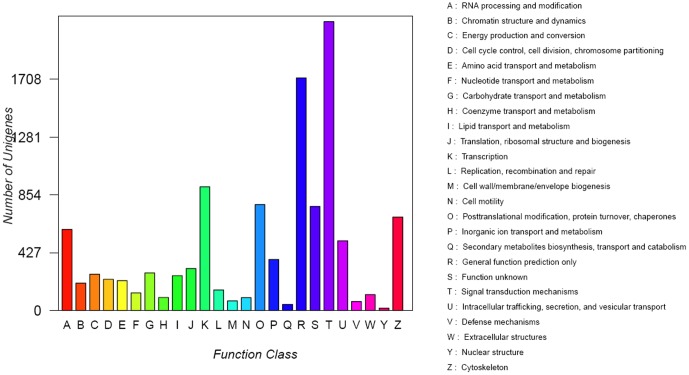
Cluster of orthologous groups (COG) classification of putative proteins.

Transcripts were also compared with the COG database, and 6,034 transcripts (2,386 genes) were matched to database entries. These transcripts were classified into 25 functional categories (Figure 3), among which the largest group (2031 transcripts) was signal transduction mechanisms, followed by general function prediction only (1718 transcripts), transcription (914 transcripts), posttranslational modification, protein turnover, and chaperones (782 transcripts). Genes matched to the nuclear structure category (17 transcripts) represented the smallest group.

**Figure 4:**
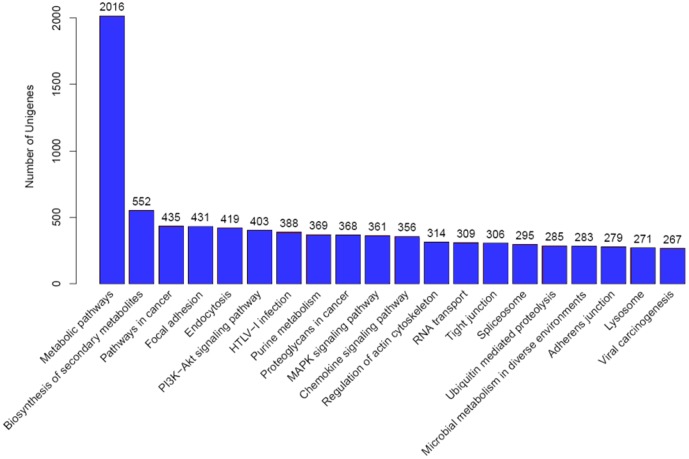
KEGG Classification of the genes. 14596 transcripts were assigned to 311 KEGG pathways. The top 20 most abundant KEGG pathways are shown.

In addition, a KEGG pathway analysis was performed on all assembled transcripts as an alternative approach for functional categorization and annotation. A total of 14,596 transcripts, corresponding to 5414 genes, were categorized into functional groups, in which the metabolism group was the most well represented, with 7821 transcripts, followed by the human disease group (7597 transcripts), organismal systems group (7179 transcripts), environmental information processing group (3460 transcripts), cellular processes group (3420 transcripts), and genetic information processing group (2481 transcripts) (Table S3 in File S1). Each functional group was made up of genes from different KEGG pathways. In addition, the number of transcripts in each KEGG pathway was counted, and the most abundant 20 KEGG pathways are shown in Figure 4. In brief, 2016 transcripts were categorized into metabolic pathways, followed by pathways for biosynthesis of secondary metabolites (552 transcripts), cancer (435 transcripts), focal adhesion (431 transcripts), and endocytosis (419 transcripts).

### Differential analysis of gene expression profiles between tissues

**Figure 5:**
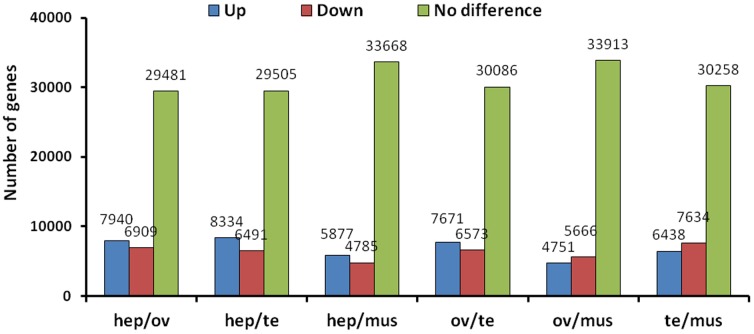
Differentially expressed genes analysis of four different crayfish tissues. hep: hepatopancreas; mu: muscle; ov: ovary; te: testis.

The expression levels of whole transcripts in the hepatopancreas, the testis, the ovary, and muscle were evaluated (Table S4 and S5 in File S1). Transcriptomic analysis of these tissues showed that more genes were overexpressed in the hepatopancreas as compared with genes expressed in the other three tissues, while more genes were underexpressed in the testis and the ovary compared with genes expressed in the hepatopancreas and muscle (Figure 5). Interestingly, although more genes were overexpressed in the hepatopancreas, fewer genes were expressed in the hepatopancreas (32999 genes) than in the muscle (37873 genes), the ovary (45083 genes), and the testis (39503 genes). This result may be due to the crucial role of the hepatopancreas in growth, which resulted in genes for metabolism being actively and highly expressed, while the ovary and the testis are reproductive organs, and thus more functional molecules are needed in reserve, but are not highly expressed.

#### Enriched pathways in the hepatopancreas

Metabolism is the basic physiological process that sustains living organisms, and it includes multiple reactions, such as the synthesis of digestive enzymes, secretion, digestion, nutrient absorption, excretion, lipid and glycogen storage, and mobilization [31]. In crustaceans, the hepatopancreas is the major metabolic organ. KEGG pathway enrichment analysis showed that regulation of amino acids, carbohydrates, lipids, and glycan metabolism were significantly enriched in the hepatopancreas compared to the other three tissues (Table 2). For example, 59 transcripts were identified in the fatty acid metabolism pathway (ko00071), and 26 of these transcripts (21 genes) were found to be significantly overexpressed in the hepatopancreas compared to muscle, including ACOX1 (acyl-CoA oxidase [EC:1.3.3.6]), ACADS (butyryl-CoA dehydrogenase [EC:1.3.8.1]), ALDH7A1 (aldehyde dehydrogenase family 7 member A1 [EC:1.2.1.3, 1.2.1.8, 1.2.1.31]), and ACAA2 (acetyl-CoA acyltransferase 2 [EC:2.3.1.16]), and only 7 transcripts (3 genes) were found to be significantly underexpressed. It was also found that the expression levels of genes in the fatty acid metabolism pathway in the hepatopancreas were significantly higher than those in the ovary and the testis, indicating that active fatty acid metabolism takes place in the hepatopancreas of *P. clarkii* (Table S6 in File S1).

**Table 2:** KEGG pathways enriched in 4 crayfish tissues with Bonferroni-corrected p-values.

tissue 1	tissue 2	#Term	Sample number	Background number	Corrected P-Value
hepato-pancreas	muscle	Lysosome	148	272	7.27E-07
		Galactose metabolism	45	66	1.96E-05
		Peroxisome	79	138	5.42E-05
		Regulation of actin cytoskeleton	169	342	5.45E-05
		Rheumatoid arthritis	39	58	0.000139
		Methane metabolism	34	49	0.000183
		PPAR signaling pathway	49	79	0.000186
		Bacterial invasion of epithelial cells	90	169	0.000266
		Starch and sucrose metabolism	64	112	0.000267
		Focal adhesion	184	389	0.000278
		Arachidonic acid metabolism	61	107	0.000422
		Basal cell carcinoma	38	60	0.000693
		Sphingolipid metabolism	56	98	0.000766
		Glutathione metabolism	58	103	0.000904
		Glycolysis/Gluconeogenesis	56	100	0.001221
		Other glycan degradation	42	71	0.001747
		Retinol metabolism	22	31	0.001909
		Metabolism of xenobiotics by cytochrome P450	37	61	0.00197
		Alanine, aspartate and glutamate metabolism	32	51	0.002217
		Pentose phosphate pathway	35	58	0.003069
		Type II diabetes mellitus	30	48	0.003427
		Shigellosis	66	128	0.005031
		Phagosome	100	208	0.005674
		Fatty acid metabolism	34	58	0.006587
		Viral myocarditis	81	165	0.007891
		Drug metabolism - other enzymes	35	61	0.008885
		Nitrogen metabolism	22	34	0.009292
		Endometrial cancer	53	102	0.012052
		Pyruvate metabolism	39	71	0.013601
		Pentose and glucuronate interconversions	24	39	0.014534
		Amino sugar and nucleotide sugar metabolism	61	124	0.026912
		Caffeine metabolism	9	11	0.028532
		Antigen processing and presentation	33	61	0.037375
		Proximal tubule bicarbonate reclamation	21	35	0.038357
		Cytokine-cytokine receptor interaction	16	25	0.044251
		Drug metabolism - cytochrome P450	30	55	0.044251
hepato-pancreas	ovary	Ribosome	85	91	0
		Lysosome	188	272	4.53E-08
		Drug metabolism - cytochrome P450	49	55	2.00E-07
		Metabolism of xenobiotics by cytochrome P450	51	61	7.90E-06
		Oxidative phosphorylation	96	135	5.70E-05
		Glutathione metabolism	76	103	6.91E-05
		Rheumatoid arthritis	44	58	0.004021
		Sphingolipid metabolism	68	98	0.004644
		Retinol metabolism	26	31	0.004991
		Lysine degradation	92	140	0.006405
		Pentose and glucuronate interconversions	31	39	0.006589
		Fatty acid metabolism	42	58	0.017066
		Ascorbate and aldarate metabolism	18	21	0.023131
		Cell cycle	116	188	0.023826
		Arginine and proline metabolism	59	88	0.023826
		Arachidonic acid metabolism	70	107	0.023826
		Parkinson's disease	82	129	0.031582
		PPAR signaling pathway	53	79	0.036184
		Melanoma	41	59	0.039756
hepato-pancreas	testis	Lysosome	163	272	4.82E-07
		Peroxisome	91	138	2.02E-06
		Amino sugar and nucleotide sugar metabolism	83	124	2.14E-06
		Fatty acid metabolism	44	58	1.18E-05
		Rheumatoid arthritis	43	58	4.29E-05
		Starch and sucrose metabolism	72	112	7.17E-05
		Oxidative phosphorylation	84	135	7.17E-05
		Metabolism of xenobiotics by cytochrome P450	44	61	7.95E-05
		Drug metabolism - cytochrome P450	40	55	0.000155
		Glutathione metabolism	65	103	0.0004
		Drug metabolism - other enzymes	42	61	0.000658
		Retinol metabolism	24	31	0.002129
		Galactose metabolism	43	66	0.003549
		Pentose and glucuronate interconversions	28	39	0.004508
		Valine, leucine and isoleucine degradation	41	63	0.004508
		Sphingolipid metabolism	59	98	0.004508
		Aminobenzoate degradation	16	19	0.004508
		Butanoate metabolism	30	43	0.004508
		Other glycan degradation	45	71	0.004508
		beta-Alanine metabolism	26	36	0.004797
		Alanine, aspartate and glutamate metabolism	34	51	0.006232
		Arginine and proline metabolism	53	88	0.007429
		DNA replication	42	67	0.008497
		PPAR signaling pathway	48	79	0.009379
		Arachidonic acid metabolism	62	107	0.009379
		Histidine metabolism	19	25	0.009451
		Cysteine and methionine metabolism	36	56	0.009451
		Two-component system	16	20	0.009557
		Nitrogen metabolism	24	34	0.010345
		Parkinson's disease	72	129	0.013287
		Antigen processing and presentation	38	61	0.014212
		Tyrosine metabolism	28	43	0.023795
		Propanoate metabolism	29	45	0.024472
		Glycosaminoglycan degradation	30	47	0.024998
		Pyrimidine metabolism	89	168	0.026852
		Glycine, serine and threonine metabolism	44	75	0.029221
		Synthesis and degradation of ketone bodies	15	20	0.032987
		Glycolysis/Gluconeogenesis	56	100	0.032987
		Tryptophan metabolism	32	52	0.035613
		Meiosis - yeast	63	115	0.035613
		Nucleotide excision repair	43	74	0.036095
		Collecting duct acid secretion	17	24	0.041595
		Pyruvate metabolism	41	71	0.048078
ovary	muscle	Ribosome	81	91	0
		Protein processing in endoplasmic reticulum	134	240	0.001548
		Type II diabetes mellitus	35	48	0.001548
		Glycolysis/Gluconeogenesis	63	100	0.001843
		DNA replication	43	67	0.014867
		Colorectal cancer	61	102	0.014867
		Insulin signaling pathway	123	230	0.019284
		Cell cycle	102	188	0.024154
		Focal adhesion	196	389	0.024154
		Mismatch repair	32	49	0.027405
		Homologous recombination	38	61	0.02987
		Fanconi anemia pathway	60	105	0.033469
		Basal cell carcinoma	37	60	0.036003
		Thyroid cancer	14	18	0.04045
		Melanoma	36	59	0.044224
		Prostate cancer	51	89	0.044446
testis	muscle	RNA polymerase	47	69	0.000652
		Focal adhesion	199	389	0.002043
		Glycolysis/Gluconeogenesis	61	100	0.002043
		Carbon fixation in photosynthetic organisms	29	42	0.007085
		Bladder cancer	19	25	0.011212
		Basal cell carcinoma	38	60	0.011212
		Cytosolic DNA-sensing pathway	30	45	0.011555
		Pyrimidine metabolism	90	168	0.018107
		Colorectal cancer	58	102	0.021629
		Regulation of actin cytoskeleton	168	342	0.03891
ovary	testis	Ribosome	80	91	0

Enriched pathways in xenobiotic metabolism, heavy metal and oxidative stress, and the innate immune system were also found in the hepatopancreas. The crayfish is well-known for its ability to survive in polluted environments, including water polluted by heavy metals, pesticides, and other chemicals, and also to tolerate hypoxia. The hepatopancreas is the primary site for accumulation and detoxification of xenobiotic pollutants in lysosomes. The R cells in the hepatopancreas perform biotransformation using enzymes, such as cytochrome p450, to sequester and detoxify xenobiotic pollutants [31]. Compared to the other tissues, several pathways were significantly enriched in the hepatopancreas: “lysosome”, “peroxisome”, “metabolism of xenobiotics by cytochrome p450”, and “drug metabolism - cytochrome P450” (Table 2). Peroxisomes are essential organelles that play key roles in redox signaling and lipid homeostasis [57]. Here, 146 transcripts were identified in the peroxisome pathway (ko04146), of which 71 transcripts were expressed at significantly higher levels in the hepatopancreas than in muscle tissue, including SOD2 (superoxide dismutase, Fe-Mn family [EC:1.15.1.1]), CAT (catalase [EC:1.11.1.6]), DDO (D-aspartate oxidase [EC:1.4.3.1]), DAO (D-amino-acid oxidase [EC:1.4.3.3]), SCP2 (sterol carrier protein 2 [EC:2.3.1.176]), and PIPOX (sarcosine oxidase/L-pipecolate oxidase [EC:1.5.3.7, 1.5.3.1]) (Figure S2, Table S7 in File S1). Only 11 transcripts were expressed at significantly lower levels in the hepatopancreas than in muscle tissue.

Methyl farnesoate and ecdysteroids are important hormones in crustaceans. Methyl farnesoate, which is synthesized in the mandibular organ (MO), is an insect juvenile hormone homologue that is believed to act as a juvenile hormone in crustaceans [58]. Juvenile hormones are involved in many biological processes, including development and reproduction. The major function of ecdysteroids is to control molting, but they are also involved in reproduction [59]. Here, genes in the insect hormone biosynthesis pathway were identified from the *P. clarkii* transcriptome. Among these genes, genes in the juvenile hormone synthesis pathway were significantly overexpressed in the hepatopancreas compared to the other three tissues (Table S8 in File S1). In particular, seven of nine transcripts that encoded a CYP15A1 (cytochrome P450, family 15, subfamily A, polypeptide 1) homologue were highly expressed in the hepatopancreas, in which the expression levels of these transcripts were more than 100-fold greater than in the other tissues. These results indicate that the hepatopancreas is an important site for genes that are responsible for the synthesis of juvenile hormone. In contrast, genes in the ecdysteroid synthesis pathways did not show the same trend, and the differences in expression of these genes were not significant between the examined tissues.

#### Enriched pathways in the ovary and the testis

The ovary and the testis are the major reproductive organs, in which the processes of oogenesis, sperm genesis, DNA replication, and meiosis occur frequently. As expected, KEGG pathway analysis showed that the pathways of “DNA replication”, “cell cycle”, “mismatch repair”, “homologous recombination”, were significantly enriched in the ovary compared to muscle and the hepatopancreas. The pathways of “DNA replication”, “pyrimidine metabolism”, “meiosis-yeast”, and “Nucleotide excision repair” were significantly enriched in the testis compared to muscle and the hepatopancreas. For example, 51 transcripts were identified in the DNA replication pathway (ko03030), of which 32 transcripts (26 genes) were expressed at significantly greater levels in the ovary than in the hepatopancreas, and of which only 2 genes were expressed at significantly lower levels in the ovary than in the hepatopancreas. Analysis showed that 26 transcripts (22 genes) were expressed at significantly higher levels in the testis than in the hepatopancreas, and only 6 transcripts (4 genes) were expressed at significantly lower levels in the testis than in the hepatopancreas (Table S9 in File S1).

In oviparous animals, vitellin is the major yolk protein that provides nutrition during embryonic development. The precursor of vitellin is vitellogenin (Vg). It is believed that extraovarian Vg is synthesized in the hepatopancreas and secreted in the hemolymph, where it is sequestered into developing oocytes by the Vg receptor (VgR) through receptor-mediated endocytosis [60]. It has been reported that multiple genes encode vitellogenin in various crustaceans, such as the shrimp *Metapenaeus ensis*, the freshwater water flea *Daphnia magna*, and the banana shrimp *Penaeus merguiensis* [61]–[63]. Here 29 transcripts (20 genes) were determined to encode vitellogenin, of which 14 transcripts were highly expressed specifically in the hepatopancreas, 6 transcripts were highly expressed specifically in the ovary, and no transcript was found to be highly expressed in the testis or in muscle. Indeed, vitellogenin was extremely difficult to detect in muscle (Table S10 in File S1), suggesting that vitellogenin is synthesized in the hepatopancreas and the ovary of *P. clarkii*. This result is consistent with previous reports in the Chinese mitten-handed crab *Eriocheir sinensis*, the tiger shrimp *Penaeus monodon,* the blue crab *Callinectes sapidus,* the freshwater crayfish *Cherax quadricarinatus*, the freshwater prawn *Macrobrachium rosenbergii*, the green mud crab *Scylla paramamosain*, and other species of shrimp and crab [64]–[68]. Interestingly, no transcript among the 20 transcripts determined to encode vitellogenin was found to be highly expressed in both the hepatopancreas and the ovary. Thus, expression of the identified vitellogenin transcripts was tissue-specific, including 14 transcripts that were hepatopancreas-specific and 6 transcripts that were ovary-specific. It has been reported that *MeVg1*, one of two vitellogenin genes in the shrimp *Metapenaeus ensis*, is expressed only in the ovary and the hepatopancreas, while the other vitellogenin gene, *MeVg2*, is expressed exclusively in the hepatopancreas [62], [69]. These results provide new insight into the expression of vitellogenin genes in the hepatopancreas and the ovary, and provide the basis for future studies on the manner in which vitellogenin genes collaboratively perform their specific functions at different developmental stages in the ovary.

The vitellogenin receptor is located in the cell membrane of oocytes and mediates vitellogenin absorption by oocytes through receptor-mediated endocytosis (RME) [70]. Unlike vitellogenin in *P. clarkii*, which was highly expressed in the hepatopancreas and in the ovary, the vitellogenin receptor gene was highly expressed in the ovary only, which is consistent with its ovary-specific expression pattern in the shrimp *Penaeus monodon* and in the freshwater prawn *Macrobrachium rosenbergii* (Table S10 in File S1) [60], [64].

#### RT-PCR Assays

**Figure 6:**
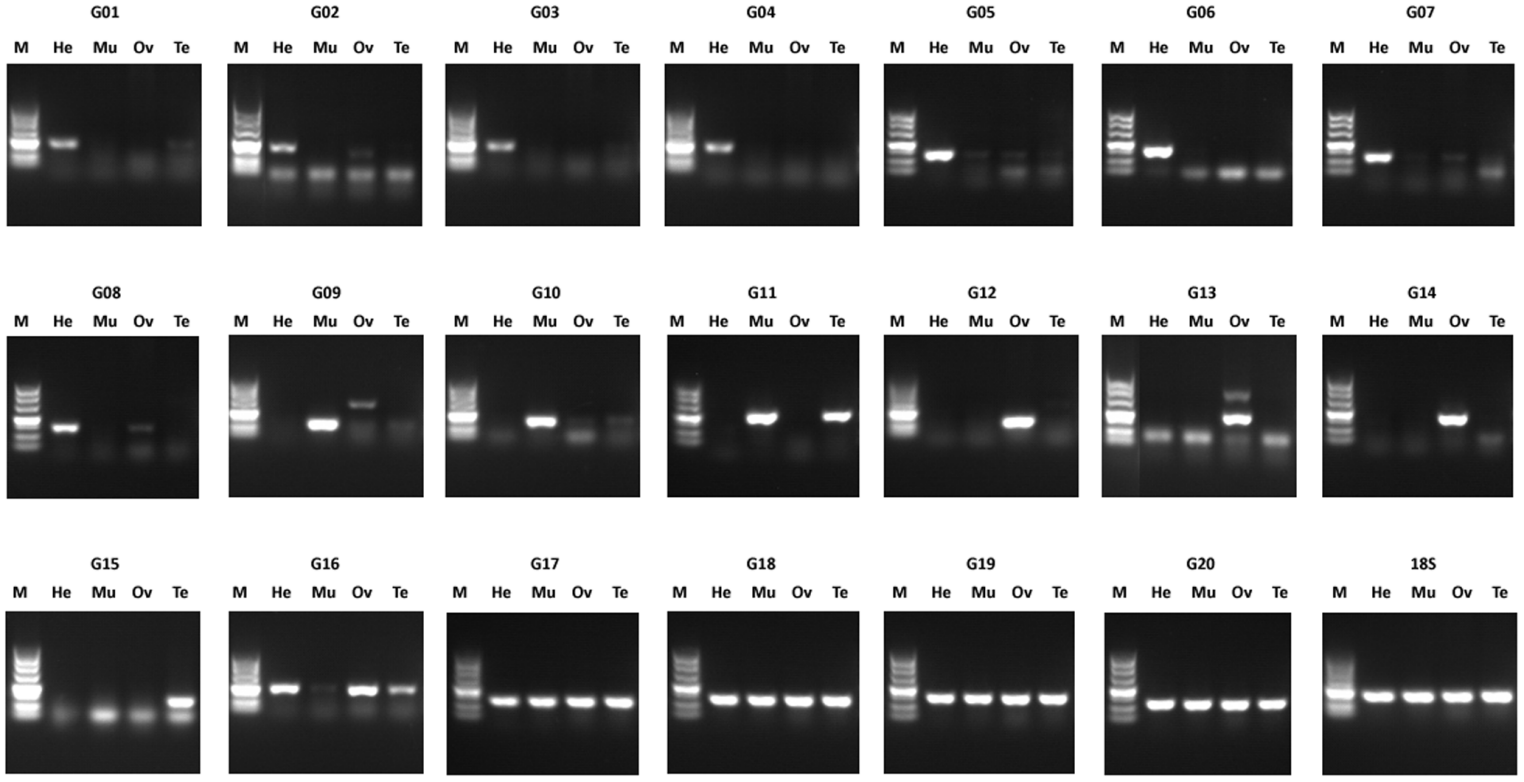
RT-PCR amplification and agarose gel (1.5%) electrophoresis of 20 transcripts. G01–G20: names of transcripts, represented transcript_ID given in Table S10 in File S1; 18S: 18S rRNA transcript; He: hepatopancreas; Mu: muscle; Ov: ovary; Te: testis; M: DNA marker.

To validate the assembled transcripts and their expression profiles in the 4 collected tissue types, 20 transcript sequences were selected for RT-PCR (reverse transcription polymerase chain reaction) amplification. Their putative gene names, primer sequences, and expected PCR product sizes are shown in Table S11 in File S1. All 20 primer pairs gave amplification products of the expected sizes (Figure 6). For G09 and G13, in addition to the expected PCR products, larger PCR unexpected products were also found in the ovary. Analysis of the FPKM levels of these 20 selected transcript sequences showed that 8 sequences (G01–G08) were specifically expressed in the hepatopancreas, 3 sequences (G09–G11) were specifically expressed in muscle, 3 sequences (G12–G14) were specifically expressed in the ovary, 1 sequence (G15) was specifically expressed in the testis, and the other 5 sequences (G16–G20) were highly expressed in 3 or 4 tissue types (Table S11 in File S1). RT-PCR analysis showed that, with the exception of sequence G11, which was indicated by FPKM analysis to be specifically expressed in muscle, but was indicated by RT-PCR to be highly expressed in both muscle and testis, the expression modes of the other 19 sequences in the 4 tissue types were consistent with their FPKM levels (Figure 6). The evaluation and validation of the assembled transcripts verified the high accuracy of Illumina paired-end sequencing and de novo assembly, and thus indicated that our study could be useful for further research into gene function.

### SNP identification

**Figure 7:**
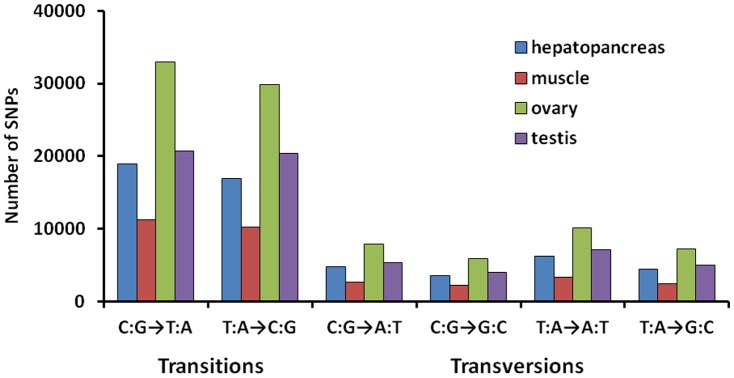
Classification of SNPs identified in the crayfish transcriptome.

Single-nucleotide polymorphisms (SNPs) are the most common type of variation in the genome. SNPs were identified by alignments of multiple sequences used for contig assembly. After excluding those that had a base mutation frequency of less than 1%, a total of 243,764 SNPs were obtained (Figure 7). The proportions of transition substitutions were 34.44% for C:G→T:A and 31.74% for T:A→C:G, compared with smaller proportions of transversion for C:G→A:T (8.49%), C:G→G:C (6.42%), T:A→A:T (11.05%) and T:A→G:C (7.86%). The total transition:transversion ratio was 1.96∶1. Differences in base structure and the numbers of hydrogen bonds between different bases resulted in a large proportion of transition type SNPs and a small proportion of transversion type SNPs. The ovary had the most SNPs (94023 SNPs), followed by the testis (62601 SNPs), hepatopancreas (54855 SNPs), and muscle (32285 SNPs). Statistics for identified SNPs in the crayfish transcriptome are shown in Figure 7.

### Microsatellite sequence identification

**Figure 8:**
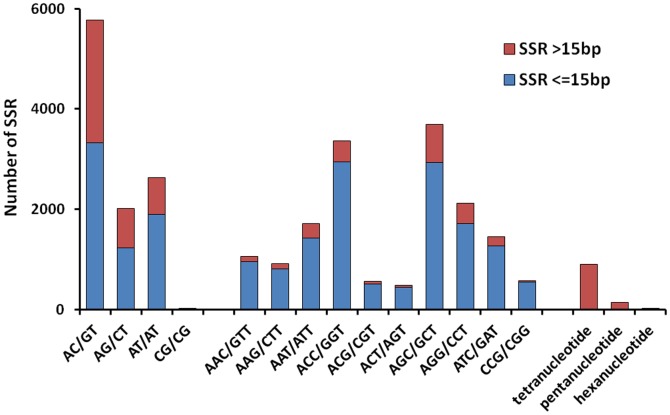
Distribution of simple sequence repeat (SSR) nucleotide classes among different nucleotide types found in the transcriptome of *P. clarkii*.

Microsatellite sequences, or simple sequence repeats (SSRs), are polymorphic loci present in genomic DNA that consist of repeated core sequences of 2–6 base pairs in length [71]. A total of 27,451 SSRs were initially identified from 29,534 transcripts, including 36.92% trinucleotide repeats, 24.14% di-nucleotide repeats, and 2.48% tetra/penta/hexanucleotide repeats (Figure 8). In addition, a total of 4775 SSRs (27.12%) were found that were more than 15 base pairs in length. Among the tri-nucleotide repeat motifs, (AGC/GCT)n (3,693 SSRs, 23.16%) and (ACC/GGT)n (3368 SSRs, 22.13%) were the most common types, and appeared significantly more than the other types of tri-nucleotide repeat motifs (Figure 8). After removing the microsatellites that lacked sufficient flanking sequences for primer design, 16953 unique sequences with microsatellites possessed sufficient flanking sequences on both sides of the microsatellites to allow the design of primers for genotyping.

## Conclusions

This is the report on the transcriptome of *P. clarkii* using *de novo* assembly techniques with next-generation sequencing. We identified 50,219 non-redundant transcripts that will provide the basis for future studies on crayfish gene function. We also explored gene expression patterns in four different tissues from *P. clarkii*, and a number of candidate novel genes were identified that may be involved in important physiological processes and are worthy of further investigation. In addition, a large number of predicted SNPs and SSRs were reported that provide a basis for further genetic analysis and crayfish breeding.

## Supporting Information

Figure S1 **The hit species distribution based on BLASTx.**
(TIF)

Figure S2 **Transcripts identified as differentially expressed between muscle tissue and the hepatopancreas in the peroxisome pathway (ko04146).** Genes for which expression levels in muscle were higher than those in the hepatopancreas are shown with a red frame, and genes for which expression levels in muscle were lower than those in the hepatopancreas are shown with a green frame.
(RAR)

File S1 **Tables S1–S11.** Table S1, Statistics for *P. clarkii* sequencing data. Table S2, Summary of BLASTX search results for the *P. clarkii* transcriptome. Table S3, Number of transcripts (genes) for each KEGG functional group. Table S4, Annotation and FPKM values for transcripts with ORFs in 4 tissue types. Table S5, Annotation and FPKM values of transcripts without ORFs in 4 tissue types. Table S6, Transcripts identified in the fatty acid metabolism pathway (ko00071) and differential expression analysis between the hepatopancreas and muscle tissue. Table S7, Transcripts identified in the peroxisome pathway (ko04146) and differential expression analysis between the hepatopancreas and muscle tissue. Table S8, Transcripts identified in the insect hormone biosynthesis pathway (ko00981). Table S9, Transcripts identified in the DNA replication pathway (ko03030) and differential expression analysis between the ovary and the hepatopancreas and between the testis and the hepatopancreas. Table S10, Transcripts encoding vitellogenin and the vitellogenin receptor identified from the *P. clarkii* transcriptome. Table S11, Information on the 20 transcript sequences selected for RT-PCR.
(XLSX)

## References

[1] Banci KRS, Viera NFT, Marinho PS, Calixto PdO, Marques OAV (2013) Predation of Rhinella ornata (Anura, Bufonidae) by the alien crayfish (Crustacea, Astacidae) *Procambarus clarkii* (Girard, 1852) in São Paulo, Brazil. Herpetology Notes 6: 339–341.

[2] Gherardi F (2006) Crayfish invading Europe: the case study of *Procambarus clarkii* . Marine and Freshwater Behaviour and Physiology 39: 175–191.

[3] Yue GH, Wang GL, Zhu BQ, Wang CM, Zhu ZY, et al (2008) Discovery of four natural clones in a crayfish species *Procambarus clarkii* . Int J Biol Sci 4: 279–282. 18781225 10.7150/ijbs.4.279 PMC2532795

[4] Wang W, Gu W, Ding Z, Ren Y, Chen J, et al (2005) A novel Spiroplasma pathogen causing systemic infection in the crayfish *Procambarus clarkii* (Crustacea: Decapod), in China. FEMS Microbiol Lett 249: 131–137. 16000238 10.1016/j.femsle.2005.06.005

[5] Cruz MJ, Rebelo R (2007) Colonization of freshwater habitats by an introduced crayfish, *Procambarus clarkii*, in Southwest Iberian Peninsula. Hydrobiologia 575: 191–201.

[6] Chen AJ, Gao L, Wang XW, Zhao XF, Wang JX (2013) SUMO-conjugating enzyme E2 UBC9 mediates viral immediate-early protein SUMOylation in crayfish to facilitate reproduction of white spot syndrome virus. J Virol 87: 636–647. 23097446 10.1128/JVI.01671-12 PMC3536383

[7] Lin LJ, Chen YJ, Chang YS, Lee CY (2013) Neuroendocrine responses of a crustacean host to viral infection: effects of infection of white spot syndrome virus on the expression and release of crustacean hyperglycemic hormone in the crayfish *Procambarus clarkii* . Comp Biochem Physiol A Mol Integr Physiol 164: 327–332. 23174320 10.1016/j.cbpa.2012.11.009

[8] Du HH, Hou CL, Wu XG, Xie RH, Wang YZ (2013) Antigenic and immunogenic properties of truncated VP28 protein of white spot syndrome virus in *Procambarus clarkii* . Fish Shellfish Immunol 34: 332–338. 23178263 10.1016/j.fsi.2012.11.007

[9] Wu XG, Xiong HT, Wang YZ, Du HH (2012) Evidence for cell apoptosis suppressing white spot syndrome virus replication in *Procambarus clarkii* at high temperature. Dis Aquat Organ 102: 13–21. 23209074 10.3354/dao02532

[10] El-Din AH, Varjabedian KG, Abdel-Gaber RA, Mohamed MM (2013) Antiviral immunity in the red swamp crayfish, *Procambarus clarkii*: hemocyte production, proliferation and apoptosis. J Egypt Soc Parasitol 43: 71–86. 23697017 10.12816/0006368

[11] Tattersall GJ, Luebbert JP, LePine OK, Ormerod KG, Mercier AJ (2012) Thermal games in crayfish depend on establishment of social hierarchies. J Exp Biol 215: 1892–1904. 22573768 10.1242/jeb.065946

[12] Buscaino G, Filiciotto F, Buffa G, Di Stefano V, Maccarrone V, et al (2012) The underwater acoustic activities of the red swamp crayfish *Procambarus clarkii* . J Acoust Soc Am 132: 1792–1798. 22978906 10.1121/1.4742744

[13] Celi M, Filiciotto F, Parrinello D, Buscaino G, Damiano MA, et al (2013) Physiological and agonistic behavioural response of *Procambarus clarkii* to an acoustic stimulus. J Exp Biol 216: 709–718. 23125346 10.1242/jeb.078865

[14] Tomina Y, Kibayashi A, Yoshii T, Takahata M (2013) Chronic electromyographic analysis of circadian locomotor activity in crayfish. Behav Brain Res 249: 90–103. 23631885 10.1016/j.bbr.2013.04.029

[15] Tierney AJ, Andrews K, Happer KR, White MK (2013) Dear enemies and nasty neighbors in crayfish: Effects of social status and sex on responses to familiar and unfamiliar conspecifics. Behav Processes. 10.1016/j.beproc.2013.06.001 23769936

[16] Ameyaw-Akumfi C, Hazlett BA (1975) Sex recognition in the crayfish *Procambarus clarkii* . Science 190: 1225–1226. 1198111 10.1126/science.1198111

[17] Araki M, Hasegawa T, Komatsuda S, Nagayama T (2013) Social status-dependent modulation of LG-flip habituation in the crayfish. J Exp Biol 216: 681–686. 23125344 10.1242/jeb.075689

[18] Leung TS, Naqvi SM, Naqvi NZ (1980) Paraquat toxicity to Louisiana crayfish (*Procambarus clarkii*). Bull Environ Contam Toxicol 25: 465–469. 7426798 10.1007/BF01985555

[19] Barbee GC, McClain WR, Lanka SK, Stout MJ (2010) Acute toxicity of chlorantraniliprole to non-target crayfish (*Procambarus clarkii*) associated with rice-crayfish cropping systems. Pest Manag Sci 66: 996–1001. 20730992 10.1002/ps.1972

[20] Al Kaddissi S, Legeay A, Elia AC, Gonzalez P, Camilleri V, et al (2012) Effects of uranium on crayfish *Procambarus clarkii* mitochondria and antioxidants responses after chronic exposure: what have we learned? Ecotoxicol Environ Saf 78: 218–224. 22154145 10.1016/j.ecoenv.2011.11.026

[21] Al Kaddissi S, Frelon S, Elia AC, Legeay A, Gonzalez P, et al (2012) Are antioxidant and transcriptional responses useful for discriminating between chemo- and radiotoxicity of uranium in the crayfish *Procambarus clarkii*? Ecotoxicol Environ Saf 80: 266–272. 22503064 10.1016/j.ecoenv.2012.03.010

[22] Bonvillain CP, Rutherford DA, Kelso WE, Green CC (2012) Physiological biomarkers of hypoxic stress in red swamp crayfish *Procambarus clarkii* from field and laboratory experiments. Comp Biochem Physiol A Mol Integr Physiol 163: 15–21. 22554447 10.1016/j.cbpa.2012.04.015

[23] Tan SH, Yuan ZD, Liu YF, Yang YN (2012) [Effects of Cd2+ on antioxidant system in hepatopancreas of *Procambarus clarkii*]. Ying Yong Sheng Tai Xue Bao 23: 2595–2601. 23286021

[24] Belfiore NM, May B (2000) Variable microsatellite loci in red swamp crayfish, *Procambarus clarkii*, and their characterization in other crayfish taxa. Mol Ecol 9: 2231–2234. 11123667 10.1046/j.1365-294x.2000.105339.x

[25] Yue GH, Li JL, Wang CM, Xia JH, Wang GL, et al (2010) High prevalence of multiple paternity in the invasive crayfish species, *Procambarus clarkii* . Int J Biol Sci 6: 107–115. 20186292 10.7150/ijbs.6.107 PMC2828620

[26] Li Y, Guo X, Cao X, Deng W, Luo W, et al (2012) Population genetic structure and post-establishment dispersal patterns of the red swamp crayfish *Procambarus clarkii* in China. PLoS One 7: e40652. 22808222 10.1371/journal.pone.0040652 PMC3393698

[27] Wu P, Qi D, Chen L, Zhang H, Zhang X, et al (2009) Gene discovery from an ovary cDNA library of oriental river prawn *Macrobrachium nipponense* by ESTs annotation. Comp Biochem Physiol Part D Genomics Proteomics 4: 111–120. 20403747 10.1016/j.cbd.2008.12.004

[28] Margulies M, Egholm M, Altman WE, Attiya S, Bader JS, et al (2005) Genome sequencing in microfabricated high-density picolitre reactors. Nature 437: 376–380. 16056220 10.1038/nature03959 PMC1464427

[29] Huse SM, Huber JA, Morrison HG, Sogin ML, Welch DM (2007) Accuracy and quality of massively parallel DNA pyrosequencing. Genome Biol 8: R143. 17659080 10.1186/gb-2007-8-7-r143 PMC2323236

[30] Novaes E, Drost DR, Farmerie WG, Pappas GJ Jr, Grattapaglia D, et al (2008) High-throughput gene and SNP discovery in Eucalyptus grandis, an uncharacterized genome. BMC Genomics 9: 312. 18590545 10.1186/1471-2164-9-312 PMC2483731

[31] Mohd-Shamsudin MI, Kang Y, Lili Z, Tan TT, Kwong QB, et al (2013) In-depth tanscriptomic analysis on giant freshwater prawns. PLoS One 8: e60839. 23734171 10.1371/journal.pone.0060839 PMC3667022

[32] Ma K, Qiu G, Feng J, Li J (2012) Transcriptome analysis of the oriental river prawn, *Macrobrachium nipponense* using 454 pyrosequencing for discovery of genes and markers. PLoS One 7: e39727. 22745820 10.1371/journal.pone.0039727 PMC3380025

[33] Li X, Cui Z, Liu Y, Song C, Shi G (2013) Transcriptome Analysis and Discovery of Genes Involved in Immune Pathways from Hepatopancreas of Microbial Challenged Mitten Crab *Eriocheir sinensis* . PLoS One 8: e68233. 23874555 10.1371/journal.pone.0068233 PMC3714283

[34] He L, Wang Q, Jin X, Wang Y, Chen L, et al (2012) Transcriptome profiling of testis during sexual maturation stages in *Eriocheir sinensis* using Illumina sequencing. PLoS One 7: e33735. 22442720 10.1371/journal.pone.0033735 PMC3307765

[35] Lemgruber Rde S, Marshall NA, Ghelfi A, Fagundes DB, Val AL (2013) Functional categorization of transcriptome in the species *Symphysodon aequifasciatus* Pellegrin 1904 (Perciformes: Cichlidae) exposed to benzo[a]pyrene and phenanthrene. PLoS One 8: e81083. 24312524 10.1371/journal.pone.0081083 PMC3849039

[36] Li E, Wang S, Li C, Wang X, Chen K, et al (2014) Transcriptome sequencing revealed the genes and pathways involved in salinity stress of Chinese mitten crab, *Eriocheir sinensis* . Physiol Genomics 46: 177–190. 24423969 10.1152/physiolgenomics.00191.2013

[37] Zeng D, Chen X, Xie D, Zhao Y, Yang C, et al (2013) Transcriptome analysis of Pacific white shrimp (*Litopenaeus vannamei*) hepatopancreas in response to Taura syndrome Virus (TSV) experimental infection. PLoS One 8: e57515. 23469011 10.1371/journal.pone.0057515 PMC3585375

[38] Chen X, Zeng D, Xie D, Zhao Y, Yang C, et al (2013) Transcriptome Analysis of *Litopenaeus vannamei* in Response to White Spot Syndrome Virus Infection. PLoS One 8: e73218. 23991181 10.1371/journal.pone.0073218 PMC3753264

[39] Sookruksawong S, Sun F, Liu Z, Tassanakajon A (2013) RNA-Seq analysis reveals genes associated with resistance to Taura syndrome virus (TSV) in the Pacific white shrimp *Litopenaeus vannamei* . Dev Comp Immunol 41: 523–533. 23921257 10.1016/j.dci.2013.07.020

[40] Li C, Weng S, Chen Y, Yu X, Lu L, et al (2012) Analysis of *Litopenaeus vannamei* transcriptome using the next-generation DNA sequencing technique. PLoS One 7: e47442. 23071809 10.1371/journal.pone.0047442 PMC3469548

[41] Li S, Zhang X, Sun Z, Li F, Xiang J (2013) Transcriptome analysis on Chinese shrimp *Fenneropenaeus chinensis* during WSSV acute infection. PLoS One 8: e58627. 23527000 10.1371/journal.pone.0058627 PMC3602427

[42] Kawahara-Miki R, Wada K, Azuma N, Chiba S (2011) Expression profiling without genome sequence information in a non-model species, Pandalid shrimp (*Pandalus latirostris*), by next-generation sequencing. PLoS One 6: e26043. 22016807 10.1371/journal.pone.0026043 PMC3189924

[43] Wang W, Wu X, Liu Z, Zheng H, Cheng Y (2014) Insights into hepatopancreatic functions for nutrition metabolism and ovarian development in the crab *Portunus trituberculatus*: gene discovery in the comparative transcriptome of different hepatopancreas stages. PLoS One 9: e84921. 24454766 10.1371/journal.pone.0084921 PMC3890295

[44] Lv J, Liu P, Gao B, Wang Y, Wang Z, et al (2014) Transcriptome Analysis of the *Portunus trituberculatus*: De Novo Assembly, Growth-Related Gene Identification and Marker Discovery. PLoS One 9: e94055. 24722690 10.1371/journal.pone.0094055 PMC3983128

[45] Cock PJ, Fields CJ, Goto N, Heuer ML, Rice PM (2010) The Sanger FASTQ file format for sequences with quality scores, and the Solexa/Illumina FASTQ variants. Nucleic Acids Res 38: 1767–1771. 20015970 10.1093/nar/gkp1137 PMC2847217

[46] Erlich Y, Mitra PP, delaBastide M, McCombie WR, Hannon GJ (2008) Alta-Cyclic: a self-optimizing base caller for next-generation sequencing. Nat Methods 5: 679–682. 18604217 10.1038/nmeth.1230 PMC2978646

[47] Grabherr MG, Haas BJ, Yassour M, Levin JZ, Thompson DA, et al (2011) Full-length transcriptome assembly from RNA-Seq data without a reference genome. Nat Biotechnol 29: 644–652. 21572440 10.1038/nbt.1883 PMC3571712

[48] Camacho C, Coulouris G, Avagyan V, Ma N, Papadopoulos J, et al (2009) BLAST+: architecture and applications. BMC Bioinformatics 10: 421. 20003500 10.1186/1471-2105-10-421 PMC2803857

[49] Conesa A, Gotz S, Garcia-Gomez JM, Terol J, Talon M, et al (2005) Blast2GO: a universal tool for annotation, visualization and analysis in functional genomics research. Bioinformatics 21: 3674–3676. 16081474 10.1093/bioinformatics/bti610

[50] Langmead B, Trapnell C, Pop M, Salzberg SL (2009) Ultrafast and memory-efficient alignment of short DNA sequences to the human genome. Genome Biol 10: R25. 19261174 10.1186/gb-2009-10-3-r25 PMC2690996

[51] Langmead B, Salzberg SL (2012) Fast gapped-read alignment with Bowtie 2. Nat Methods 9: 357–359. 22388286 10.1038/nmeth.1923 PMC3322381

[52] Reiner A, Yekutieli D, Benjamini Y (2003) Identifying differentially expressed genes using false discovery rate controlling procedures. Bioinformatics 19: 368–375. 12584122 10.1093/bioinformatics/btf877

[53] Robinson MD, Smyth GK (2007) Moderated statistical tests for assessing differences in tag abundance. Bioinformatics 23: 2881–2887. 17881408 10.1093/bioinformatics/btm453

[54] Li H (2011) A statistical framework for SNP calling, mutation discovery, association mapping and population genetical parameter estimation from sequencing data. Bioinformatics 27: 2987–2993. 21903627 10.1093/bioinformatics/btr509 PMC3198575

[55] Koboldt DC, Chen K, Wylie T, Larson DE, McLellan MD, et al (2009) VarScan: variant detection in massively parallel sequencing of individual and pooled samples. Bioinformatics 25: 2283–2285. 19542151 10.1093/bioinformatics/btp373 PMC2734323

[56] Faircloth BC (2008) msatcommander: detection of microsatellite repeat arrays and automated, locus-specific primer design. Mol Ecol Resour 8: 92–94. 21585724 10.1111/j.1471-8286.2007.01884.x

[57] Rottensteiner H, Theodoulou FL (2006) The ins and outs of peroxisomes: co-ordination of membrane transport and peroxisomal metabolism. Biochim Biophys Acta 1763: 1527–1540. 17010456 10.1016/j.bbamcr.2006.08.012

[58] Nagaraju GPC (2007) Is methyl farnesoate a crustacean hormone? Aquaculture 272: 39–54.

[59] Nagaraju GP (2011) Reproductive regulators in decapod crustaceans: an overview. J Exp Biol 214: 3–16. 21147963 10.1242/jeb.047183

[60] Tiu SH, Benzie J, Chan SM (2008) From hepatopancreas to ovary: molecular characterization of a shrimp vitellogenin receptor involved in the processing of vitellogenin. Biol Reprod 79: 66–74. 18218613 10.1095/biolreprod.107.066258

[61] Tokishita S, Kato Y, Kobayashi T, Nakamura S, Ohta T, et al (2006) Organization and repression by juvenile hormone of a vitellogenin gene cluster in the crustacean, *Daphnia magna* . Biochem Biophys Res Commun 345: 362–370. 16681994 10.1016/j.bbrc.2006.04.102

[62] Tsang WS, Quackenbush LS, Chow BK, Tiu SH, He JG, et al (2003) Organization of the shrimp vitellogenin gene: evidence of multiple genes and tissue specific expression by the ovary and hepatopancreas. Gene 303: 99–109. 12559571 10.1016/s0378-1119(02)01139-3

[63] Phiriyangkul P, Puengyam P, Jakobsen IB, Utarabhand P (2007) Dynamics of vitellogenin mRNA expression during vitellogenesis in the banana shrimp Penaeus (*Fenneropenaeusmerguiensis*) using real-time PCR. Mol Reprod Dev 74: 1198–1207. 17342737 10.1002/mrd.20629

[64] Revathi P, Iyapparaj P, Munuswamy N, Krishnan M (2012) Vitellogenesis during the ovarian development in freshwater female prawn *Macrobrachium rosenbergii* (De Man). International Journal of Aquatic Science 3: 13–27.

[65] Jia X, Chen Y, Zou Z, Lin P, Wang Y, et al (2013) Characterization and expression profile of Vitellogenin gene from *Scylla paramamosain* . Gene 520: 119–130. 23466977 10.1016/j.gene.2013.02.035

[66] Ferre LE, Medesani DA, Garcia CF, Grodzielski M, Rodriguez EM (2012) Vitellogenin levels in hemolymph, ovary and hepatopancreas of the freshwater crayfish *Cherax quadricarinatus* (Decapoda: Parastacidae) during the reproductive cycle. Rev Biol Trop 60: 253–261. 22458222 10.15517/rbt.v60i1.2759

[67] Zmora N, Trant J, Chan SM, Chung JS (2007) Vitellogenin and its messenger RNA during ovarian development in the female blue crab, *Callinectes sapidus*: gene expression, synthesis, transport, and cleavage. Biol Reprod 77: 138–146. 17409377 10.1095/biolreprod.106.055483

[68] Li K, Chen L, Zhou Z, Li E, Zhao X, et al (2006) The site of vitellogenin synthesis in Chinese mitten-handed crab *Eriocheir sinensis* . Comp Biochem Physiol B Biochem Mol Biol 143: 453–458. 16480909 10.1016/j.cbpb.2005.12.019

[69] Tiu SH, Hui JH, He JG, Tobe SS, Chan SM (2006) Characterization of vitellogenin in the shrimp *Metapenaeus ensis*: expression studies and hormonal regulation of MeVg1 transcription in vitro. Mol Reprod Dev 73: 424–436. 16425293 10.1002/mrd.20433

[70] Roth Z, Khalaila I (2012) Identification and characterization of the vitellogenin receptor in *Macrobrachium rosenbergii* and its expression during vitellogenesis. Mol Reprod Dev 79: 478–487. 22674884 10.1002/mrd.22055

[71] Queller DC, Strassmann JE, Hughes CR (1993) Microsatellites and kinship. Trends Ecol Evol 8: 285–288. 21236170 10.1016/0169-5347(93)90256-O

