# Supplementary figures and images for: In-Depth Transcriptome Analysis of the Red Swamp Crayfish Procambarus clarkii
Source: PLoS One. 2014 Oct 22;9(10):e110548. doi: 10.1371/journal.pone.0110548 (PMC4206422; doi:10.1371/journal.pone.0110548)

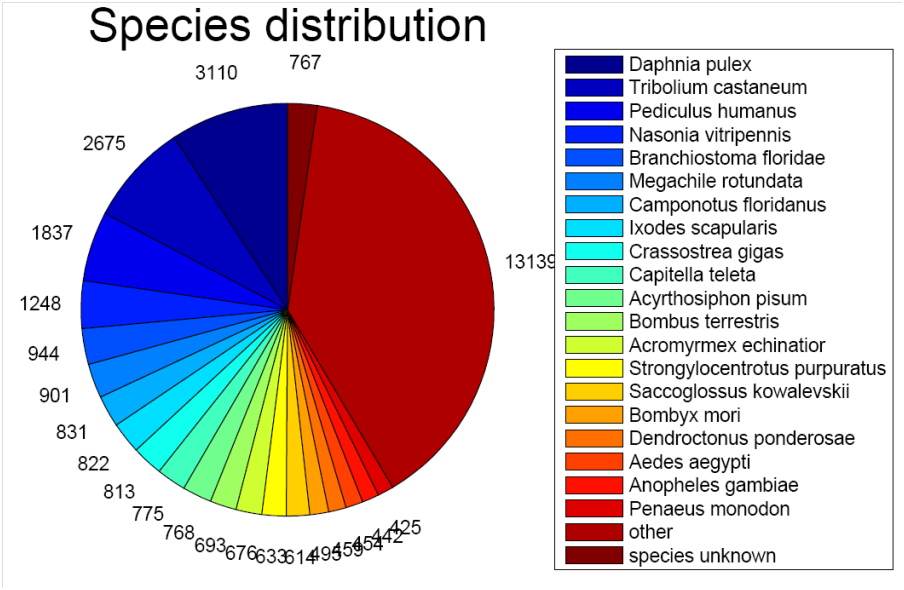

Supplement: Figure S1 — The hit species distribution based on BLASTx. (TIF) [file pone.0110548.s001.tif]
